# Proactive infection control measures to prevent hospital outbreaks in a regional hospital in Hong Kong: approaching zero outbreaks in a 5-year time period

**DOI:** 10.1186/2047-2994-4-S1-P233

**Published:** 2015-06-16

**Authors:** VC Cheng, JW Tai, K-Y Yuen

**Affiliations:** 1 Infection Control Unit, Queen Mary Hospital, Hong Kong; 2 Microbiology, The University of Hong Kong, Hong Kong, Hong Kong

## Introduction

Hospital outbreaks of epidemiologically important pathogens are usually caused by lapses in infection control measures and result in increased morbidity, mortality and cost. However, there is no benchmark to compare the occurrence of hospital outbreaks across hospitals.

## Objectives

It is worth considering setting up such surveillance data for hospital outbreaks, similar to surgical site and device related infections as found in the National Healthcare Safety Network (NHSN) report and International Nosocomial Infection Control Consortium (INICC) respectively.

## Methods

We implemented proactive infection control measures with an emphasis on timely education of healthcare workers and hospitalized patients about directly observed hand hygiene on outbreak prevention in Queen Mary Hospital (QMH), a teaching hospital. Our benchmarked performance (outbreak episodes per 1 million patient discharges and 1 million patient days) was compared with those of other regional public hospitals without these measures between 2010 and 2014.

## Results

During the study period, QMH only had 1 hospital outbreak, resulting in 1.48 and 0.45 outbreak episodes per 1 million patient discharges and patient days respectively, which were significantly lower than the corresponding overall rates in 7 acute regional hospitals (24.26 and 6.70 outbreak episodes per 1 million patient discharges and patient days respectively, p<0.001) and that of all 42 public hospitals in Hong Kong (41.62 and 8.65 outbreak episodes per 1 million patients discharges and patients days respectively, p<0.001).

## Conclusion

This large study on benchmarked rate of hospital outbreaks per patient discharges or patient days suggested that proactive infection control interventions may minimize the risk of hospital outbreaks.

## Disclosure of interest

None declared.

